# Status of Aluminum Oxide Gate Dielectric Technology for Insulated-Gate GaN-Based Devices

**DOI:** 10.3390/ma15030791

**Published:** 2022-01-21

**Authors:** Anthony Calzolaro, Thomas Mikolajick, Andre Wachowiak

**Affiliations:** 1 Nanoelectronics, TU Dresden, D-01062 Dresden, Germany; thomas.mikolajick@tu-dresden.de; 2 NaMLab gGmbH, Nöthnitzer Str. 64a, D-01187 Dresden, Germany; andre.wachowiak@namlab.com

**Keywords:** GaN, gate dielectric, aluminum oxide, interface, traps, instability

## Abstract

Insulated-gate GaN-based transistors can fulfill the emerging demands for the future generation of highly efficient electronics for high-frequency, high-power and high-temperature applications. However, in contrast to Si-based devices, the introduction of an insulator on (Al)GaN is complicated by the absence of a high-quality native oxide for GaN. Trap states located at the insulator/(Al)GaN interface and within the dielectric can strongly affect the device performance. In particular, although AlGaN/GaN metal–insulator–semiconductor high electron mobility transistors (MIS-HEMTs) provide superior properties in terms of gate leakage currents compared to Schottky-gate HEMTs, the presence of an additional dielectric can induce threshold voltage instabilities. Similarly, the presence of trap states can be detrimental for the operational stability and reliability of other architectures of GaN devices employing a dielectric layer, such as hybrid MIS-FETs, trench MIS-FETs and vertical FinFETs. In this regard, the minimization of trap states is of critical importance to the advent of different insulated-gate GaN-based devices. Among the various dielectrics, aluminum oxide (Al_2_O_3_) is very attractive as a gate dielectric due to its large bandgap and band offsets to (Al)GaN, relatively high dielectric constant, high breakdown electric field as well as thermal and chemical stability against (Al)GaN. Additionally, although significant amounts of trap states are still present in the bulk Al_2_O_3_ and at the Al_2_O_3_/(Al)GaN interface, the current technological progress in the atomic layer deposition (ALD) process has already enabled the deposition of promising high-quality, uniform and conformal Al_2_O_3_ films to gate structures in GaN transistors. In this context, this paper first reviews the current status of gate dielectric technology using Al_2_O_3_ for GaN-based devices, focusing on the recent progress in engineering high-quality ALD-Al_2_O_3_/(Al)GaN interfaces and on the performance of Al_2_O_3_-gated GaN-based MIS-HEMTs for power switching applications. Afterwards, novel emerging concepts using the Al_2_O_3_-based gate dielectric technology are introduced. Finally, the recent status of nitride-based materials emerging as other gate dielectrics is briefly reviewed.

## 1. Introduction

Owing to the large bandgap of 3.43 eV, resulting in a high electric breakdown field of 3.3 MV/cm and in a low intrinsic carrier concentration, and to the large saturation velocity of 2.5 × 10^7^ cm/s, GaN is one of the most promising semiconductors for the future energy-efficient generation of high-power, high-frequency and high-temperature electronics [1,2,3,4]. Besides the unique intrinsic material properties, one of the most attractive properties of GaN is the possibility to exploit the polar nature of GaN-based materials to form AlGaN/GaN heterostructures featuring a two-dimensional electron gas (2DEG) at the heterointerface with a high carrier density of over 1 × 10^13^ cm^−2^ and high mobility values exceeding 2000 cm^2^ V^−1^ s^−1^ [5,6]. AlGaN/GaN heterostructures enable the fabrication of high electron mobility transistors (HEMTs) which can significantly outperform the traditional Si power devices in terms of breakdown strength, on-resistance and switching speed, achieving higher power density and higher energy efficiency [7,8].

Nowadays, GaN-on-Si HEMTs qualified for 200 V and 650 V high voltage power switching applications with operating frequency capabilities in the MHz range are commercially available and on the way towards 1.2 kV applications using engineered substrates [1,9,10]. For targeting higher voltage capabilities up to 1.7–1.8 kV, current aperture vertical electron transistors (CAVETs) adopting AlGaN/GaN heterojunctions have also recently attracted significant attention [11,12,13], where the high conductivity of the 2DEG channel is combined with the better field distribution of the vertical device geometry, and hence with the capability of vertical architectures of achieving an even higher breakdown voltage without enlarging the device area, in contrast to lateral transistors. In addition, GaN-based HEMTs with downscaled gate lengths to the sub-100 nm regime have also been demonstrated to achieve maximum current gain cutoff frequencies over 200 GHz, which are well suited for radio frequency (RF) high power amplifiers for 5G and beyond applications [14,15,16,17,18].

Despite the potentiality of AlGaN/GaN HEMTs, one of the most serious problems degrading the device performance and reliability is represented by the exceedingly high leakage currents through the Schottky-gate contact, especially under forward gate bias, which limits the gate voltage swing and the maximum on-state current of the device, resulting in reduced power efficiency and weak device failure protection [19]. In particular, a small gate swing is a strong limiting factor for power switching applications due to faulty gate voltage overshoots often occurring in circuits, which can eventually lead to early device failures. Moreover, since GaN-based HEMTs are naturally normally on (or depletion-mode) transistors with a negative threshold voltage (V_th_), normally off (or enhancement-mode) HEMTs with a positive V_th_ are highly preferred to guarantee safe operation and for the reduced power consumption in power switching devices [20,21]. However, since normally off devices require a large positive gate voltage to be turned on, the problem of gate leakage currents becomes even more critical in normally off HEMTs. Similarly, in RF applications, power amplifiers using Schottky-gate HEMTs can suffer from reduced gain and efficiency caused by large gate inputs, which can drive the devices into deep forward bias regimes [22].

The employment of a metal–insulator–semiconductor (MIS) gate is an efficient way to suppress the gate leakage currents of AlGaN/GaN HEMTs, enabling reduced power consumption, a larger gate bias swing and a better immunity to gate breakdown [23,24,25]. However, in contrast to Si-based devices, the introduction of an insulator in AlGaN/GaN metal–insulator–semiconductor high electron mobility transistors (MIS-HEMTs) is complicated by the absence of a high-quality native oxide for (Al)GaN. Trap states located at the dielectric/(Al)GaN interface or within the dielectric can lead to dynamic charge/discharge processes, which are especially critical in the case of wide bandgap GaN-based materials where the traps can be deeply located in the bandgap and can cause severe operational instability due to their slow detrapping behavior [26,27,28,29]. The instability of the threshold voltage in AlGaN/GaN MIS-HEMTs is one of the major challenges [30,31,32,33]. In particular, a serious V_th_ shift induced by the “spill-over” of electrons from the 2DEG channel towards the dielectric/(Al)GaN interface in forward gate bias conditions has often been reported [34,35,36,37]. Another problem is the degradation of the current linearity in the transfer characteristics of AlGaN/GaN MIS-HEMTs, which can be responsible for gain loss and the degradation of large signal linearity in power amplifiers [38].

**Figure 1:**
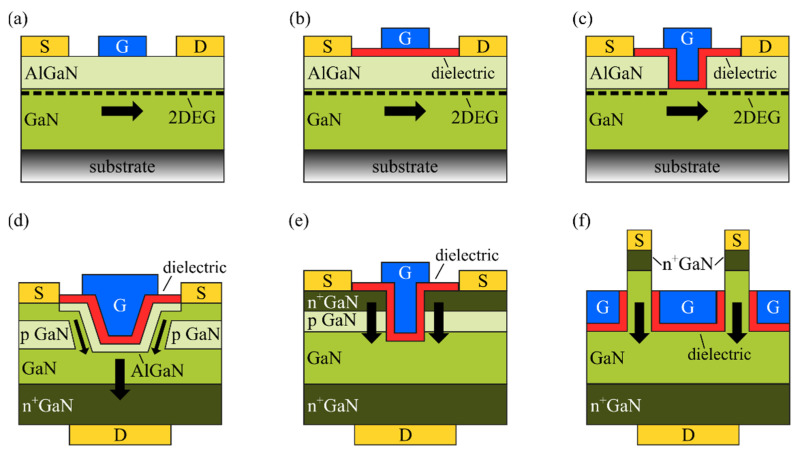
Schematic of representative GaN-based transistors: (**a**) Schottky-gate HEMT; (**b**) MIS-HEMT; (**c**) hybrid MIS-FET with fully recessed AlGaN barrier; (**d**) trench CAVET; (**e**) trench MIS-FET; (**f**) vertical FinFET.

Besides insulated-gate GaN-based transistors adopting AlGaN/GaN heterostructures, other emerging GaN-based devices in the form of MIS-FETs use a gate dielectric layer on a GaN channel, such as lateral hybrid GaN MIS-FETs [39], vertical GaN trench MIS-FETs [40] and vertical FinFETs [41]. Figure 1 schematically summarizes the main configurations of lateral and vertical GaN-based transistors employing a gate dielectric layer. A Schottky-gate HEMT is reported for comparison in Figure 1a. Hybrid GaN MIS-FETs obtained by a fully recessed AlGaN barrier layer are especially attractive for normally off operation and large gate voltage operation [42], while GaN trench MIS-FETs have drawn attention among other vertical transistor concepts since they are inherently normally off with a V_th_ > 3 V and do not need the regrowth of the AlGaN/GaN channels [43,44]. Similar to MIS-HEMTs, instabilities over the gate dielectric affecting the device performance can arise in GaN devices with MIS-FET configurations due to the trap states at the dielectric/GaN interface influencing the V_th_ and reducing the current drive capability or/and bulk or border traps within the dielectric itself, which might mostly affect the long-term reliability performance of the device [43,45]. Moreover, differently from the MIS-HEMTs, where the 2DEG formed at the AlGaN/GaN interface benefits from the spatial separation from the dielectric/AlGaN interface, minimizing the interface scattering processes [46], interface traps in GaN MIS-FET configurations are located in the proximity of the electron channel and are more prone to act as impurity scattering centers, additionally affecting the carrier mobility [47]. This is particularly challenging for transistor concepts including a recess of the AlGaN barrier layer, as in the case of trench MIS-FETs and vertical FinFETs, since the etching process can critically affect the properties of the dielectric/GaN interface [48].

Therefore, regardless of the transistor concept and design, trap states need to be minimized to ensure the safe operation and long-term lifetime of the insulated-gate GaN-based transistors. In particular, a gate dielectric technology aiming to improve the dielectric/(Al)GaN interface and dielectric bulk quality is essential to enhance the performance of the device. In general, various insulator materials have been employed, with SiO_2_, SiN and Al_2_O_3_ as the most commonly used dielectrics [7,29,49,50]. The same dielectric layer deposited both underneath the gate as well as between the gate and the ohmic contacts of the source and drain usually functions both as the gate dielectric and the passivation layer [51]. The latter has been indeed reported to mitigate the effects of drain current collapse and leakage currents at the (Al)GaN surface due to the passivation of trap states at the surface [52,53,54]. However, even though excellent device characteristics have been obtained, trap states in MIS gate structures still remain one of the biggest challenges for insulated-gate GaN-based transistors, and the practical implementation of these devices has been hindered by the concerns over the gate dielectric stability and reliability [1].

In this paper, the current status of the gate dielectric technology employing Al_2_O_3_ for insulated-gate GaN-based transistors is reviewed. First, the relevant aspects taken into account for selecting a suitable gate dielectric for GaN-based transistors are highlighted and the influence of this additional layer on the device parameters and performance is discussed. Afterwards, the state of the art of Al_2_O_3_ as a gate dielectric is presented with a particular attention to the recent progress in engineering high-quality Al_2_O_3_/(Al)GaN interfaces and to the performance of Al_2_O_3_-gated GaN-based MIS-HEMTs for power switching applications. Novel emerging concepts using the Al_2_O_3_-based gate dielectric technology are also introduced. Finally, the recent status of nitride-based materials emerging as other gate dielectrics is briefly reviewed.

## 2. Gate Dielectrics on (Al)GaN

The design of a MIS gate structure for insulated-gate GaN-based transistors requires consideration of the properties of the bandgap, the band offset to (Al)GaN, the permittivity and the chemical stability of the insulators [7,29,49,50,51]. For a sufficient suppression of the gate leakage currents, even at forward gate bias operation, a large bandgap material as well as large band offsets to (Al)GaN are necessary, in particular for power switching devices. On the other hand, a high value of permittivity is favorable to obtain high transconductance [55]. In particular, in the case of MIS-HEMTs, since the introduction of a dielectric leads to a reduction of the gate-to-channel capacitance with respect to Schottky-gate HEMTs, a high permittivity dielectric reduces the capacitive contribution of the gate dielectric, enabling it to obtain a stronger coupling between the gate and the 2DEG channel, and hence to maintain a high transconductance, which is especially important for RF devices. At the same time, in normally on MIS-HEMTs, high-permittivity materials can minimize the shift of the threshold voltage towards negative values when compared to Schottky-gate HEMTs, which is beneficial to reduce the static power consumption and to improve the energy efficiency of the device [7].

**Figure 2:**
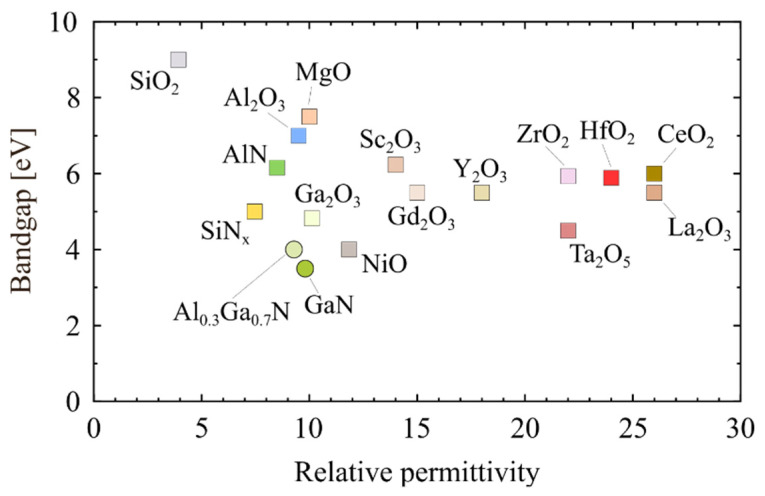
Energy bandgap versus permittivity for major insulators and GaN compounds. Data taken from [7,29,49,50,51].

**Figure 3:**
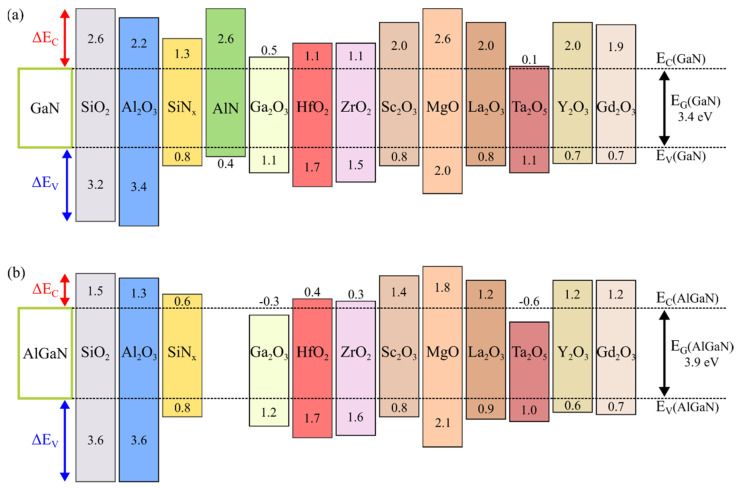
Conduction band offset (∆E_C_) and valence band offset (∆E_V_) of various dielectric materials with respect to (**a**) GaN, calculated by Robertson and Falabretti [56], and to (**b**) Al_0.3_Ga_0.7_N, calculated by Reddy et al. [57]. Note that, in (**a**,**b**), the different energy bandgaps of the insulators were assumed in the calculations. The conduction band (E_C_) and valence band (E_V_) of GaN and AlGaN are marked as dashed lines. The energy bandgap (E_G_) of GaN and AlGaN is also indicated.

Various insulator materials have already been considered as gate dielectrics in insulated-gate GaN-based transistors. Figure 2 reports the relationship between the bandgap and permittivity for the relevant insulators and nitride compounds. Figure 3a shows the band offsets of the insulators on the GaN as calculated by Robertson and Falabretti, who first predicted the band alignment of the GaN and the insulators based on the calculation of the charge neutrality levels (E_CNL_) [56]. The band offsets of the dielectrics on Al_0.3_Ga_0.7_N, recently determined by Reddy et al. using the same method, are illustrated in Figure 3b [57]. Note that, as shown from the comparison of Figure 3a,b, the different values of the energy bandgap of the same insulators are used in the calculations performed by Robertson and Falabretti [56] and by Reddy et al. [57].

SiO_2_ is an attractive insulator due to its large bandgap, large band offset to (Al)GaN and chemical stability. In fact, after Khan and coworkers first applied SiO_2_ to AlGaN/GaN MIS-HEMTs to control the gate leakage currents and improve the gate voltage swing capability [24], further high-performance MIS-HEMTs using SiO_2_ have been demonstrated [58,59]. Nevertheless, the relatively low dielectric constant of SiO_2_ represents a disadvantage compared to other dielectrics. From this perspective, various high-permittivity dielectrics such as HfO_2_, ZrO_2_, Ta_2_O_5_, La_2_O_3_, CeO_2_, TiO_2_, etc., have been applied to the MIS gate structures of GaN HEMTs [60,61,62,63,64,65,66,67,68,69,70,71,72]. Although higher g_m_ values have been achieved in some cases, most of these insulators have reported to be relatively susceptible to leakage problems due to the relatively small band offsets with respect to (Al)GaN [49,68,73,74]. Similar observations of high gate leakage currents were reported for MIS gate structures employing dielectrics such as SiN_x_ and Ga_2_O_3_ due to the small conduction band offsets [51,75,76,77]. Ga_2_O_3_ would be appealing as a native oxide grown by thermal or chemical processes. However, in addition to the small band offset to GaN, Ga_2_O_3_ grown by thermal oxidation at low temperatures has a slow growth rate, while surface damage can be caused at higher growth temperatures [51]. Moreover, the growth of Ga_2_O_3_ is even more difficult on AlGaN since Al is more easily oxidized than Ga. Differently, SiN_x_ deposited by in situ metal organic chemical vapor deposition (MOCVD) or by low-pressure chemical vapor deposition (LPCVD) has emerged as a promising candidate as a gate dielectric as well as a passivation layer [42,78]. Similarly, AlN has also been reported in a few studies to be suitable as a gate insulator and passivation layer, especially due to its small lattice mismatch to (Al)GaN [51,79,80,81]. Other attempts have also used dielectrics like NiO, MgO and Sc_2_O_3_ [82,83,84,85,86,87], stacked dielectric layers like SiN_x_/SiO_2_, SiN_x_/Al_2_O_3_ and HfO_2_/Al_2_O_3_ [88,89,90] or engineered alloys such as SiON, HfSiO_x_ and LaLuO_3_ in order to tune the dielectric constant and band gap of the insulators [90,91,92,93]. A comprehensive overview and comparison of the various insulators which have been considered as gate dielectrics for insulated-gate GaN-based devices is given in [7,29,49,50,51].

Among the insulators, Al_2_O_3_ remains one of the most attractive insulators as a gate dielectric because of its large bandgap and conduction band offset to (Al)GaN, relatively high permittivity (~9) as well as high breakdown field (~10 MV/cm) and thermal and chemical stability against (Al)GaN [75,94,95]. Additionally, the considerable technological progress in the atomic layer deposition (ALD) process enables the deposition of high-quality Al_2_O_3_ films to the gate structures in GaN transistors. In the next section, the status of the gate dielectric technology using Al_2_O_3_ for GaN-based devices is reviewed.

## 3. Al_2_O_3_ for Insulated-Gate GaN Devices

Table 1 reports the physical parameters of the energy bandgap (E_G_), conduction band offset (∆E_C_) and valence band offset (∆E_V_) obtained experimentally from amorphous Al_2_O_3_ films deposited on GaN and AlGaN by various deposition methods. Note that the bandgap of the amorphous Al_2_O_3_ ranges between 6.7 eV and 7.6 eV depending on the method of the oxide film growth, and it is lower than the value for the crystalline bulk α-Al_2_O_3_ (8.8 eV–9 eV) considered in the theoretical calculations (Figure 3). In fact, it is well known that the E_G_ of Al_2_O_3_ compounds strongly depends on its crystallographic phase [96,97]. Momida et al. investigated the structure of amorphous Al_2_O_3_ by first-principles calculations, concluding that the reduction of the bandgap of amorphous Al_2_O_3_ compared to crystalline Al_2_O_3_ could be related to the changes in the density of the Al_2_O_3_ compounds and the average coordination number of Al atoms [98]. Toyoda et al. showed that annealing at temperatures of 800 °C led to phase transformations of the Al_2_O_3_ films from amorphous to crystalline, which correlated to a significant increase in the energy bandgap and the modification of the conduction band discontinuity [99]. Afanas’ev et al. pointed out that for Al_2_O_3_ films treated at temperatures above 800 °C, the widening of the Al_2_O_3_ bandgap with the phase transformation from amorphous to crystalline mostly occurred at the valence band side [96,97]. Differently, Yang et al. revealed that the annealing processes at a lower temperature of 650 °C can affect the band bending of GaN but has almost no effect on the Al_2_O_3_/GaN band offset [100]. The decrease of the bandgap of amorphous Al_2_O_3_ has also been associated with defect-induced states located in the bandgap [101]. This could explain the large discrepancy between the theoretical (Figure 3) and experimental (Table 1) values of ∆E_V_. In fact, since in the case of Al_2_O_3_ the valence band maximum states are associated with the O 2p states, and the conduction band minimum states are associated with the Al 3s, 3p states [102], the rehybridization between Al 3s, 3p and O 2p modifies the charge transfer between Al and O and consequently decreases the bandgap, thus increasing the valence band maximum [51]. In contrast to ∆E_V_, the experimental values of ∆E_C_ obtained for the Al_2_O_3_/(Al)GaN system are consistent with the theoretical predictions and make Al_2_O_3_ a suitable dielectric for insulated-gate GaN-based transistors.

In addition to the physical properties of the bandgap of Al_2_O_3_ and the band offsets in the Al_2_O_3_/(Al)GaN system, high-quality dielectric layers in terms of defects and bulk traps and an Al_2_O_3_/(Al)GaN interface with a low interface trap density are required to deliver a high performance and highly efficient MIS gate structure, as discussed above. It is important to mention that these properties strongly depend on the deposition technique and temperature, the crystalline structure of the film and the surface and annealing treatments [51]. Among the techniques explored for the deposition of Al_2_O_3_ films, such as sputtering [103], the oxidation of a thin Al layer [53] and MOCVD [104,105,106], the ALD technique is widely used. The main advantages of the ALD method are the low deposition temperature (<350 °C), the excellent film thickness control as well as the high uniformity and conformality, which have enabled the deposition of high-quality Al_2_O_3_ films and Al_2_O_3_/(Al)GaN interfaces compared to other methods. Nevertheless, despite substantial progress in the ALD technology, large amounts of defects in the as-deposited Al_2_O_3_ bulk material and interface traps at the Al_2_O_3_/(Al)GaN interface are still present and still hinder the success of the insulated-gate GaN devices [1].

**Table 1:** Energy bandgap (E_G_), conduction band offset (∆E_C_) and valence band offset (∆E_V_) measured for Al_2_O_3_ films on GaN and AlGaN. The deposition method is reported in the second column, where ALD = atomic layer deposition; PEALD = plasma-enhanced atomic layer deposition; CVD = chemical vapor deposition; MBD = molecular beam epitaxy; ECR = electron cyclotron resonance. In addition, the measurement method is noted in column 3, where C–V = capacitance–voltage measurements; F–N = Fowler–Nordheim characteristics; IPE = internal photoemission; XPS = X-ray photoelectron spectroscopy; UPS = ultraviolet photoelectron spectroscopy; XAS = X-ray absorption spectroscopy.

Structure	Deposition Method	Measurement Method	E_G_ (eV)	∆E_C_ (eV)	∆E_V_ (eV)	Ref.
Al_2_O_3_/GaN	ALD	C–V	-	-	1.2	[107]
Al_2_O_3_/GaN	ALD	XPS and F–N plot	6.7	2.2	-	[108]
Al_2_O_3_/GaN	ALD	XPS	6.6	2.0	1.2	[109]
Al_2_O_3_/GaN	ALD	IPE and C–V	-	2.2	-	[110]
Al_2_O_3_/GaN	PEALD	XPS and UPS	6.7	2.1	1.2	[111]
Al_2_O_3_/GaN	PEALD	XPS and UPS	-	1.3	1.8	[100]
Al_2_O_3_/GaN	CVD	XPS and XAS	7.6	2.7	1.5	[99]
Al_2_O_3_/Al_0.3_Ga_0.7_N	MBD + ECR plasma oxidation	XPS	7.0	2.1	0.8	[53,75]
Al_2_O_3_/Al_0.25_Ga_0.75_N	ALD	XPS	6.9	1.8	1.2	[112]
Al_2_O_3_/Al_0.25_Ga_0.75_N	ALD	XPS	6.7	1.8	0.9	[26]

### 3.1. Al_2_O_3_/(Al)GaN Structures

The presence of defects acting as traps or fixed charge centers within the Al_2_O_3_ films and at the Al_2_O_3_/(Al)GaN interface is of critical importance because of their potential to affect the threshold voltage and the gate leakage currents of the MIS gate structures [51], eventually deteriorating the operational stability and the reliability of the insulated-gate GaN-based devices.

For ALD-Al_2_O_3_/(Al)GaN structures, a positive fixed charge arising from donor-type interface states and/or defect levels in the bulk Al_2_O_3_ was often reported [112,113,114,115]. In this regard, Esposto et al. [107] and Son et al. [116] pointed out that fixed charges at the Al_2_O_3_/GaN interface shifted the flat-band voltage (V_FB_) in the C–V curves of Al_2_O_3_/GaN capacitors. A shift of the V_FB_ towards the negative bias direction in Al_2_O_3_/GaN structures was observed by Kaneki et al. [115]. Similar shifts in the C–V characteristics attributed to interface states acting as fixed charges were reported for Al_2_O_3_/AlGaN/GaN structures by Mizue et al. [26] and Yatabe et al. [73]. Nishiguchi et al. [38] reproduced the observed negative shift in the C–V curve of Al_2_O_3_/AlGaN structures, assuming an effective fixed positive charge of +1.2 × 10^13^ cm^−2^ in the Al_2_O_3_ layer or at the Al_2_O_3_/AlGaN interface.

In line with this, annealing treatments have been reported to affect the V_FB_ and V_th_ of Al_2_O_3_/(Al)GaN structures as a result of a change in the defect levels in Al_2_O_3_ films [117,118]. For example, Hashizume et al. [114] reported a V_FB_ recovery of Al_2_O_3_/GaN structures after a postmetallization annealing (PMA) in N_2_ at 200–400 °C, possibly attributed to the reduction of the donor-type interface states and/or the defect levels in the bulk. Similarly, Hung et al. [119] obtained a V_FB_ recovery by PMA in H_2_/N_2_ forming gas at 400–550 °C. Zhou et al. [120] showed a permanent positive shift of the V_th_ in ALD-Al_2_O_3_-gated MIS-HEMTs after a postdeposition annealing (PDA) at 600 °C in N_2_, which was also suggested to be caused by a reduction of the deep-level bulk or interface traps. For similar reasons, a recovery of the V_th_ of MIS-HEMTs towards positive bias values was reported by Nishiguchi et al. [38] when using a reverse-bias anneal at 300 °C in air, and by Nakazawa et al. [121] with an anneal process at 750 °C in O_2_ atmosphere.

The exact nature of the fixed charges in the bulk of the as-deposited Al_2_O_3_ or in the vicinity of the Al_2_O_3_/(Al)GaN interfaces is still under debate, with native defects in the oxide layer or dangling bonds at the interface being the major candidates. Choi et al. [122] investigated the impact of native point defects in Al_2_O_3_ by first-principle calculations, revealing that oxygen vacancies introduce charge-state transition levels near the GaN conduction band edge, which can act as border traps close to the Al_2_O_3_/n-GaN interface or as source of leakage current through the dielectric. However, other defects such as aluminum vacancies and interstitials have been identified to act as fixed-charge centers [122]. Weber et al. [123] also suggested that aluminum vacancy and oxygen interstitial defects introduce negatively charged centers while the aluminum interstitials act as positively charged centers, affecting carrier scattering in the channel and the threshold voltage of the device. Moreover, Liu et al. [124] studied the energy levels of the oxygen vacancy in Al_2_O_3_. Shin et al. [125] and Kim et al. [126] identified oxygen and Al dangling bonds as the origin of the fixed charges in ALD-Al_2_O_3_. Huang et al. [127] suggested that these defective dangling bonds, which are also associated to fixed positive charges and acceptor-like border traps, can be suppressed by the substitution of H_2_O as an oxygen source with O_3_ for the ALD deposition of Al_2_O_3_. Other groups have also demonstrated the influence of using different ALD precursors and different deposition temperatures on oxide charges, as well as the interface traps of Al_2_O_3_ films [128,129,130,131,132].

Defect states inside Al_2_O_3_ can affect the leakage current of the MIS gate structures through trap-assisted tunneling mechanisms. For Al_2_O_3_-gated MIS-HEMTs under forward bias, Liu et al. [133] and Yoshitsugu et al. [131] showed that trap-assisted tunneling (TAT) and Poole–Frenkel emissions (PFE) are dominant at medium electric fields and temperatures above 0 °C, whereas Fowler–Nordheim tunneling (FNT) dominates at high electrical fields and temperatures below 0 °C. In addition, Yoshitsugu et al. [131] estimated a TAT-related trap energy of about 1.0 eV below the conduction band minimum of Al_2_O_3_. Wu et al. [134] instead suggested that TAT is the dominant transport mechanism in high oxide fields, with trap energies of ~1.1–1.2 eV, while PFE was responsible for medium oxide field gate current transport. Recently, Heuken et al. [135] also suggested that the time-dependent dielectric breakdown (TDDB) of ALD-Al_2_O_3_ films occurs with the presence of an initial defect density in the film and is then related to the formation of a percolation path by randomly generated defects in the oxide under stress bias. The time to breakdown was found to be thermally activated, with an activation energy of 1.25 eV, similar to the reported values of the activation energy of TAT in Al_2_O_3_ at a high oxide field [131,134].

**Figure 4:**
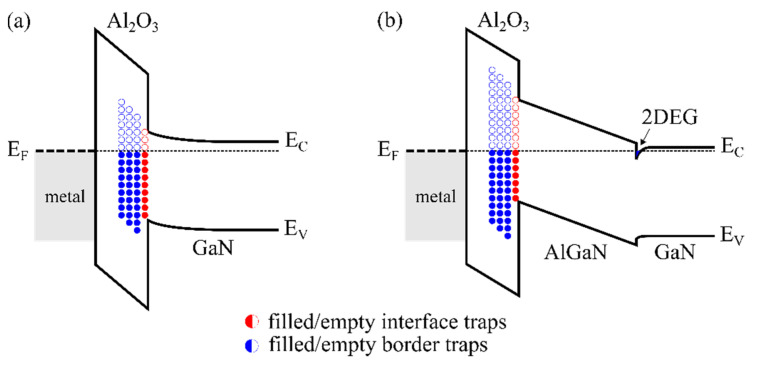
Schematic band diagram of the (**a**) Al_2_O_3_/GaN structure and (**b**) Al_2_O_3_/AlGaN/GaN heterostructure at equilibrium, showing border traps near the Al_2_O_3_/(Al)GaN interface and interface traps at the Al_2_O_3_/(Al)GaN interface. E_C_ and E_V_ are the conduction and valence bands of (**a**) GaN and (**b**) AlGaN, respectively. E_F_ denotes the Fermi energy.

While defect states and bulk traps acting as fixed charges mostly affect the absolute value of the threshold voltage, the charging and discharging of bulk traps, especially border traps near the Al_2_O_3_/(Al)GaN interface, and interface traps deeply located in the bandgap of the (Al)GaN at the Al_2_O_3_/(Al)GaN interface can induce significant dynamic instabilities of the threshold voltage and of the drain current during device operation due to their slow detrapping behavior. A schematic illustration of the band diagram of Al_2_O_3_/GaN and Al_2_O_3_/AlGaN/GaN structures, including border and interface traps, is shown in Figure 4.

**Figure 5:**
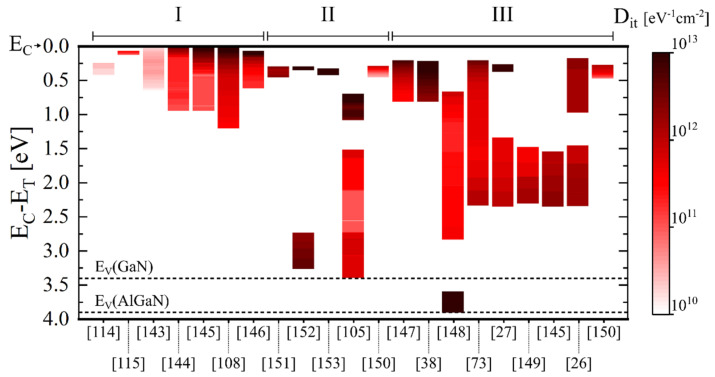
Interface density distributions (column) extracted from literature for (I) Al_2_O_3_/GaN, (II) Al_2_O_3_/GaN/Al_x_Ga_1x_N/GaN and (III) Al_2_O_3_/Al_x_Ga_1−x_N/GaN structures. The corresponding reference is indicated at the bottom of the graph for each column. The conduction band minimum E_C_ of GaN and AlGaN is set at 0 eV as reference. The valence band maximum E_V_ of GaN and AlGaN, accordingly to the bandgap values of 3.4 eV and 3.9 eV, respectively, are also illustrated as dashed lines.

For these reasons, many groups have focused their efforts on the characterization and minimization of trap states at the dielectric/(Al)GaN interface of the MIS gate structures. Figure 5 illustrates a summary of the interface trap density (D_it_) distributions reported in the literature for Al_2_O_3_/(Al)GaN structures. Note that the best results reported in each reference have been illustrated in Figure 5. The Terman method [136] and conductance method [137] are often used to estimate the interface trap state densities of Al_2_O_3_/GaN structures. Differently, since for Al_2_O_3_/AlGaN/GaN structures the evaluation of interface trap states is more challenging due to the presence of a double interface (Al_2_O_3_/AlGaN and AlGaN/GaN) complicating the potential distribution over the structure, more advanced techniques such as conductance dispersion techniques [138,139,140] and frequency and/or temperature-dependent capacitance voltage measurements [26,73,141] are employed. More detailed overviews on the characterization of the electronic states at the insulator/(Al)GaN interfaces of GaN-based MIS-HEMTs with respect to their applicability and potential limitations are given by Ramanan et al. [142] and Yatabe et al. [49].

The results reported in Figure 5 highlight the presence of high-density interface trap states, especially at energies close to the conduction and valence band edges of (Al)GaN. For Al_2_O_3_/GaN interfaces, minimum values of the interface state densities in the range of 10^10^–10^11^ cm^−2^ eV^−1^ have been reported [114,115,143,144,145,146]. In comparison, Al_2_O_3_/AlGaN interfaces have shown minimum values of interface state densities that are about one order of magnitude higher [26,27,38,73,145,147,148,149,150]. Mizue et al. [26] suggested that this difference can be due to oxygen incorporation into AlGaN or to a higher density of defects in the AlGaN layer. Note also that some groups investigated Al_2_O_3_/GaN/AlGaN/GaN structures where a thin GaN layer (~1–3 nm) was present on top of the AlGaN layer, possibly affecting the distribution of the interface trap states [105,150,151,152,153]. A very thin GaN cap layer is indeed often included in the AlGaN/GaN epitaxial material, as it also helps to protect the AlGaN surface and to reduce leakage currents. Gregušová et al. [150] obtained an interface trap state density that was two to three times lower for the Al_2_O_3_-gated AlGaN/GaN structures with a GaN cap compared to ones without a GaN cap. On the contrary, Ťapajna et al. [106] reported almost the same C–V characteristics and interface trap state distributions for Al_2_O_3_/(GaN)/AlGaN/GaN structures with and without a GaN cap layer. For ALD-Al_2_O_3_/AlGaN/GaN structures, Mizue et al. [26] estimated the trap states density distribution at the ALD-Al_2_O_3_/AlGaN interface for the first time, showing that trap states with densities higher than 1 × 10^12^ cm^−2^ eV^−1^ exist at the Al_2_O_3_/AlGaN interface. To evaluate the near-midgap electronic states at room temperature (RT), a photoassisted C–V method using photon energies less than the AlGaN bandgap was developed [26,73]. For states close to the valence band of (Al)GaN, Matys et al. [154,155] developed a method based on the measurement and simulations of the photo-capacitance of MIS gate heterostructures. Combining this method with the photoassisted capacitance–voltage technique, the interface state density in the entire band gap at the Al_2_O_3_/AlGaN interface was determined, revealing the presence of a large amount of trap states with D_it_ values higher than 1 × 10^13^ cm^−2^ eV^−1^ also near the valence band edge [148].

When using Al_2_O_3_ films on (Al)GaN, particular attention has to be given to the temperature processes applied after the dielectric deposition. Hori et al. [108] showed that the annealing process at 800 °C for the ohmic contact formation applied after the ALD-Al_2_O_3_ deposition created a large number of microcrystalline regions in the Al_2_O_3_ layer, causing a pronounced increase of the leakage current of the Al_2_O_3_/n-GaN structures. To prevent this effect, an “ohmic-first” approach with a SiN protection layer was applied, which maintained the amorphous phase in the atomic configuration of Al_2_O_3_, leading to a sufficient suppression of the leakage current. In addition, protecting the surface with a SiN layer during annealing resulted in the low interface trap densities of less than 1 × 10^12^ cm^−2^ eV^−1^ extracted from the C–V characteristics of the Al_2_O_3_/GaN structures.

Other processing steps for the fabrication of GaN devices are also critical and can affect the interface quality and the electrical properties of the Al_2_O_3_/(Al)GaN structures. To achieve normally off operation, recessed gates are often employed in MIS-HEMTs or hybrid MIS-FETs. For this reason, the influence of inductively coupled plasma (ICP) etching on the interface properties of Al_2_O_3_/(Al)GaN structures has also been investigated. Yatabe et al. [73] estimated the state density distribution at the Al_2_O_3_/AlGaN interface of MIS structures subjected to ICP dry etching of the AlGaN surface, using for the first time the combination of the photoassisted C–V method and the modeling of the C–V curves [26,156]. Trap state densities higher than 2 × 10^12^ cm^−2^ eV^−1^ were obtained at the Al_2_O_3_/AlGaN interface of the ICP-etched structures [73]. Without the ICP etching of AlGaN, a near-midgap D_it_ of about 1 × 10^12^ cm^−2^ eV^−1^ or less was obtained. Similarly, Kim et al. [144] also investigated the effects of a Cl_2_-based ICP etching on the interface properties of Al_2_O_3_/GaN structures. From the X-ray photoelectron spectroscopy (XPS) and transmission electron microscopy (TEM) analyses, it was shown that the ICP etching caused a disorder of the chemical bonds at the GaN surface. This resulted in high-density trap states with a density larger than 1 × 10^13^ cm^−2^ eV^−1^ near the conduction band edge of the GaN at the Al_2_O_3_/GaN interface, which was suggested to include defects related to nitrogen vacancy (V_N_) levels. A decrease of the interface state density was obtained by applying a PDA process in N_2_ at 400 °C, which partially recovered the V_N_-related levels, thus increasing the chemical bond order at the GaN surface. Yatabe et al. [149] also reported that the ICP etching of the AlGaN surface introduced a monolayer-level crystalline roughness, the disorder of the chemical bonds and various types of defect complexes including V_N_, resulting in high trap state densities of up to 8 × 10^12^ cm^−2^ eV^−1^ at the Al_2_O_3_/AlGaN interface. Fang et al. [157] also reported that Cl_2_-based ICP etching enhanced the deep centers at the GaN surface originating from V_N_ and other defect complexes.

Other studies have demonstrated the importance of PDA and PMA treatments to minimize the interface trap states at the Al_2_O_3_/(Al)GaN interface. From the TEM investigations, Hashizume et al. [114] revealed that PMA in N_2_ at 300–400 °C led to a uniform distribution of the lattice constant near the interface of the ALD-Al_2_O_3_/GaN MIS structures, which resulted in excellent C–V characteristics almost without frequency dispersion and a reduced D_it_ ranging from 1 to 4 × 10^10^ cm^−2^ eV^−1^ at energies near the conduction band edge. Similar values of D_it_ at the Al_2_O_3_/GaN interface after PMA in N_2_ at 400 °C were also very recently obtained by Ando et al. [158]. Ando et al. [147] also demonstrated that a PMA in N_2_ at 300 °C led to a similar reduction of the electronic states at the ALD-Al_2_O_3_/AlGaN interface. Kaneki et al. [115] pointed out that annealing under reverse bias at 300 °C in air for 3 h is also beneficial to decrease the interface state density of ALD-Al_2_O_3_/GaN structures, and it is more effective than PDA in N_2_ at 400–700 °C, probably due to a relaxation of the dangling bonds and/or the point defects at the GaN surface. Moreover, almost no shift of the V_FB_ with respect to the expected value was observed in the C–V curves due to the reduction of the donor-type interface states and/or defect levels in the bulk Al_2_O_3_. Similar effects of the reverse-bias annealing were obtained by Nishiguchi et al. [38] for ALD-Al_2_O_3_/AlGaN structures. Winzer et al. [143] reported that PDA in O_2_ or forming gas (H_2_/N_2)_ at 500 °C were more efficient for decreasing the traps at the Al_2_O_3_/GaN interface than PDA in N_2_ at the same temperature. A very low interface trap density of less than 5 × 10^11^ cm^−2^ eV^−1^ was achieved for Al_2_O_3_/GaN structures treated by forming gas PDA at 500 °C. However, it was also reported that forming gas PDA resulted in a detrimental increase of the leakage currents of the Al_2_O_3_ films. Similar results were reported by Long et al. [159], where the effect of trap passivation during the forming gas anneal was correlated to the incorporation of hydrogen at the interface.

Similar to annealing processes, surface treatments are also effective in reducing interface trap states at the Al_2_O_3_/(Al)GaN interface. Hori et al. [27,145] demonstrated that an N_2_O-radical treatment can decrease interface states both at the Al_2_O_3_/GaN and Al_2_O_3_/AlGaN interfaces. For Al_2_O_3_/AlGaN structures, the interface state density was estimated to be 1 × 10^12^ cm^−2^ eV^−1^ or less around the midgap and 8 × 10^12^ cm^−2^ eV^−1^ near the conduction band edge [27]. Calzolaro et al. [151] recently reported a significant reduction of frequency dispersion of the C–V characteristics of Al_2_O_3_/GaN/AlGaN/GaN structures after a remote O_2_ plasma-based surface treatment prior to the ALD-Al_2_O_3_ deposition combined with a PMA in N_2_ at 350 °C. The D_it_ was estimated to be reduced to a value in the order of 2 × 10^12^ cm^−2^ eV^−1^ near the conduction band edge.

Trapping mechanisms at the Al_2_O_3_/(Al)GaN interface are especially critical for AlGaN/GaN MIS-HEMTs under forward gate bias, where electrons can spill over from the 2DEG channel towards the dielectric by overcoming the AlGaN barrier and become trapped at the Al_2_O_3_/(Al)GaN interface [34,35,36,37]. Similarly, charge trapping in high-density electronic states at the interface has been reported to lead to a significant screening of the gate electric field and the consequent loss of control of the surface potential of the barrier layer, causing the degradation of the current linearity and the saturation of the current at forward bias in AlGaN/GaN MIS-HEMTs [38]. In this regard, the next section focuses on reviewing the recent progress on the performance of Al_2_O_3_-gated MIS-HEMTs.

### 3.2. Al_2_O_3_-Gated MIS-HEMTs

Among the issues facing the MIS gate toward the improvement of the performance of AlGaN/GaN MIS-HEMTs, the dynamic V_th_ instability caused by the trapping mechanisms involving the gate dielectric is the one major concern [1]. The instability of the V_th_ has been reported under various bias conditions [31,32,34,78,160,161]. In particular, the large V_th_ shift induced by forward gate bias stress due to electron trapping at the dielectric/(Al)GaN interface is one of the most serious problems for the operational stability and reliability of the device [34,35,36,37]. For this reason, many groups have focused their efforts on studying the origin of the V_th_ instability and various fabrication processing strategies to overcome this issue.

For Al_2_O_3_-gated MIS-HEMTs, Lu et al. [32] reported that a larger V_th_ shift towards the forward bias direction was induced by increasing the gate positive bias stress in the pulsed current-voltage (I-V) measurements. Similar results were obtained by other groups [28,31,35,151,160,162,163]. Bisi et al. [160] pointed out that the large positive shift of the V_th_ can also promote the current collapse of MIS-HEMTs. Regarding the origin of the V_th_ instability, Ťapajna et al. [105] discussed the effect of interface states and bulk traps on the V_th_ shift in Al_2_O_3_-gated MIS-HEMTs. Wu et al. [153] and Zhu et al. [33] pointed out that the V_th_ shift during a positive gate bias stress was highly correlated to the trap states at the dielectric/(Al)GaN interface but also to the border traps near the interface. Fixed charges within the dielectric are also involved in the V_th_ shift mechanism [107,116].

**Figure 6:**
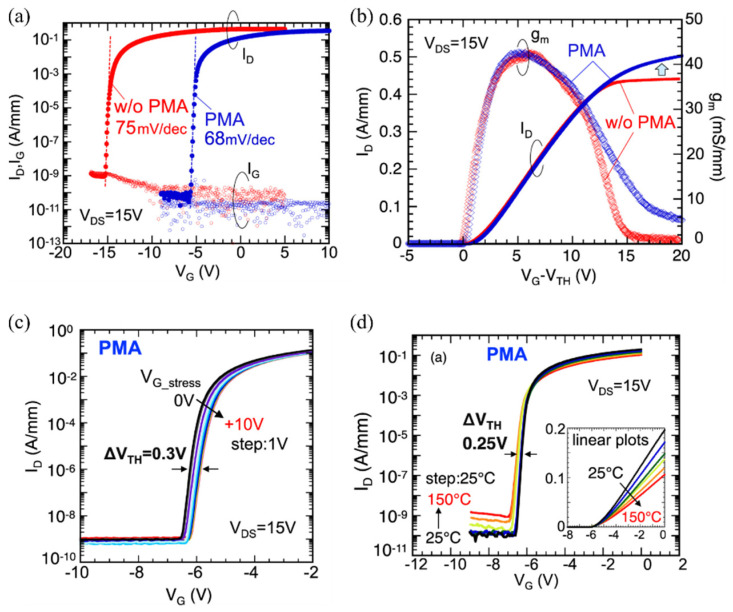
Transfer characteristics of Al_2_O_3_-gated AlGaN/GaN MIS-HEMTs fabricated on free-standing HVPE GaN substrates and subjected to PMA at 300 °C in N_2_ atmosphere, reported by Ando et al. [147]. In (**a**,**b**), the transfer characteristics of MIS-HEMTs with and without PMA are compared in a semi-log scale and as a function of the gate overdrive voltage, respectively. Transfer curves in (**c**,**d**) were obtained after applying an initial gate voltage stress up to 10 V and by increasing the temperature up to 150 °C, respectively.

A reduction of the interface and/or border traps by means of annealing and surface treatments can lead to an improvement of the dynamic V_th_ instability of MIS-HEMTs. In addition, as mentioned before, the current linearity and the saturation of current at forward bias of MIS-HEMTs can be also affected by a change in the density of the electronic states at the dielectric/(Al)GaN interface [38]. Hori et al. [27] reported that the reduction of the interface states obtained by applying an N_2_O-radical treatment on the AlGaN surface prior to the ALD-Al_2_O_3_ deposition led to a higher maximum drain current of the MIS-HEMTs at the positive gate bias and a suppressed V_th_ instability under the negative gate bias stress even at 150 °C. Nishiguchi et al. [38] showed that the improvement of the Al_2_O_3_/AlGaN interface by the reverse-bias anneal at 300 °C in air for 3 h of Al_2_O_3_-gated MIS-HEMTs gave a better gate control of the current even at forward gate bias, effectively enhancing the current linearity, subthreshold behavior and the maximum drain current of the device. Moreover, reduced gate leakage currents and more stable V_th_ under forward bias stress and at higher temperatures were obtained. Similarly, Ando et al. [147] recently reported on the improved gate controllability and current linearity of MIS-HEMTs with the Al_2_O_3_ gate dielectric as a result of a reduction of the electronic states at the Al_2_O_3_/AlGaN interface after PMA in N_2_ at 300 °C. A subthreshold slope of 68 mV dec^−1^ and excellent V_th_ and operation stability up to 150 °C were also achieved, as shown in Figure 6. Note that in this case Ando et al. [147] pointed out that the improvement of the device performance also benefited from using epitaxial GaN layers grown on free-standing GaN substrates from hydride vapor phase epitaxy (HVPE) with a low dislocation density. Very recently, Calzolaro et al. [151] reported that the reduction of interface trap states by a remote O_2_ plasma-based surface treatment before the ALD-Al_2_O_3_ deposition combined with a PMA in N_2_ at 350 °C resulted in a better V_th_ stability in pulsed I-V measurements. It is worth mentioning that, despite the benefits of the PMA treatments, specific attention has to be paid to the employment of higher PMA temperatures, as it can affect the gate leakage currents of the devices using ALD-grown Al_2_O_3_ films [119,164]. Therefore, a trade-off must be considered when using the PMA treatment between the quality of the Al_2_O_3_/(Al)GaN interface and the gate leakage currents in a certain voltage range of operation.

As in the case of the surface and annealing treatments, various strategies in the fabrication process of the devices can also be adopted to influence the trap states at the interface and, therefore, suppress the V_th_ instability. Szabó et al. [31] reported that for MIS-HEMTs where the deposition of the Al_2_O_3_ gate dielectric was performed before the ohmic contacts formation and at annealing temperature of 650 °C resulted in an improvement of the V_th_ stability compared to devices where the Al_2_O_3_ was deposited after the ohmic contacts formation was obtained with a high temperature anneal of 850 °C. It was suggested that this result was a consequence of a better Al_2_O_3_/(Al)GaN interface quality. Nakazawa et al. [165] applied an interesting approach based on the selective area regrowth of AlGaN to reduce the impact on the ALD-Al_2_O_3_/AlGaN interface of the dry etching process used for the fabrication of normally off AlGaN/GaN MIS-HEMTs with recessed gate structures. With this approach, they reported a reduced V_th_ instability compared to Al_2_O_3_-gated MIS-HEMTs with dry-etched recessed gates.

Trapping mechanisms related to the gate dielectric can lead to the failure of the device. For this reason, reliability tests of the gate dielectric are also essential to bring the MIS-HEMT devices to industrial maturity. In this regard, Meneghesso et al. [30] performed an extensive analysis of trapping mechanisms and the reliability issues of AlGaN/GaN MIS-HEMTs using different insulators. They reported a significant correlation between the dynamic V_th_ shift and gate leakage currents under forward gate bias stress and suggested that trapping effects were determined by the electrons trapped in the gate insulator or at the AlGaN/insulator interface. Wu et al. [166] investigated the positive bias temperature instability (PBTI) in hybrid GaN MIS-FETs. Since the defect distribution inside the ALD-Al_2_O_3_ was found to be centered at about 1.15 eV away from the conduction band of the GaN with a narrow spread in energy, the ALD-Al_2_O_3_ gate dielectric was suggested to be very promising to improve the PBTI reliability. Meneghesso et al. [30] also measured the TDDB characteristics of MIS-HEMTs with Al_2_O_3_ as gate dielectrics. Since the time-to-failure of devices indicated a Weibull distribution with slopes larger than 1.0, they demonstrated high robustness for ALD-Al_2_O_3_. Similarly, a Weibull distribution with a slope of 2.87 was extracted from the TDDB measurements of the Al_2_O_3_-gated MIS structures by Wu et al. [134]. Huang et al. [127] also achieved good TDDB behavior and a high breakdown electric field of 8.5 MV cm^−1^ in recessed-gate MIS-HEMTs with a gate dielectric stack consisting of 13 nm of ALD-Al_2_O_3_ deposited using O_3_ as an oxygen source and grown on top of 2 nm of ALD-Al_2_O_3_ deposited using a H_2_O oxygen source. For the ALD-Al_2_O_3_ films on the GaN, Kachi et al. [167] reported a TDDB lifetime at RT and 150 °C of more than 20 years at an electric field of 3 MV cm^−1^. Kikuta et al. [168] obtained a time-to-breakdown for the ALD-Al_2_O_3_ on a dry-etched GaN of more than 40,000 years at 3 MV cm^−1^ and RT. In contrast, a time-to-breakdown of only 10^2^–10^3^ s was obtained at 250 °C, which was suggested to be caused by large TAT leakage currents.

As mentioned before, the dielectric layer employed in MIS-HEMTs can be used both as a gate dielectric and a passivation layer to reduce current collapse. Hashizume et al. [53,75] first demonstrated the use of an Al_2_O_3_ layer as a gate dielectric and a passivation scheme to control the current collapse in AlGaN/GaN HEMTs. Moreover, comparing the effects of surface passivation on MIS-HEMTs and Schottky-gate HEMTs, Tajima and Hashizume [169] showed a more pronounced reduction of the current collapse in Al_2_O_3_-gated MIS-HEMTs in contrast to Schottky-gated HEMTs, with Al_2_O_3_ serving only as a surface passivation. The suppression of the current collapse with a passivation layer, arising from negative surface charges, injected from gate edges to surface states was generally attributed to a reduction of electronic states at the AlGaN surface and of the peak field near the gate edge. Park et al. [94] reported for the first time on the use of Al_2_O_3_ deposited by ALD as a gate dielectric and passivation layer for AlGaN/GaN MIS-HEMTs. Park et al. [94] and Ye et al. [23] reported on the excellent electrical characteristics of AlGaN/GaN MIS-HEMTs using ALD-Al_2_O_3_ as a gate dielectric and passivation layer. Despite the improvements obtained by Al_2_O_3_-based passivation schemes for MIS-HEMT devices, further work is still required to limit and fully understand the current collapse phenomena in GaN transistors [1]. A more detailed overview about surface passivation for GaN-based transistors can be found in [29,49,50,51].

### 3.3. Modified Al_2_O_3_ Gate Dielectrics

Besides the use of pure Al_2_O_3_ films, other approaches involving the use of Al_2_O_3_-based bilayer gate stack dielectrics, interface engineering techniques or Al_2_O_3_-based compound materials have been investigated to combine the properties of Al_2_O_3_ with the favorable properties of other dielectric materials.

Kambayashi et al. [170] applied a SiO_2_/Al_2_O_3_ gate stack (layers indicated from top to bottom) in hybrid GaN MIS-FETs, thus demonstrating a high-performance device with a channel mobility of 192 cm^2^/Vs. Using a SiO_2_/Al_2_O_3_ gate stack, Guo and del Alamo [171,172] studied the origin of PBTI and negative bias temperature instability (NBTI) in hybrid GaN MIS-FETs. It was shown that for a composite SiO_2_/Al_2_O_3_ gate oxide, the resulting V_th_ shifts are due to electron trapping or detrapping in pre-existing oxide traps and the generation of oxide traps near the oxide/GaN interface. Van Hove et al. [173] applied an ALD-Al_2_O_3_/in situ MOCVD-Si_3_N_4_ gate bilayer stack in AlGaN/GaN MIS-HEMTs to achieve excellent electrical device characteristics with lower gate leakage currents, more stable threshold voltages and reduced current collapse when compared to Al_2_O_3_-gated MIS-HEMTs. Capriotti et al. [174] investigated the fixed interface charges between the AlGaN and the Al_2_O_3_/in situ SiN gate stack of AlGaN/GaN MIS-HEMTs. Colon and Shi [90] fabricated AlGaN/GaN MIS-HEMTs with low gate leakage currents using an ALD-HfO_2_/Al_2_O_3_ bilayer stack as well as an ALD-HfAlO_x_ ternary compound as gate dielectrics to achieve a higher dielectric constant than Al_2_O_3_ and a higher conduction band offset, thermal stability and crystallization temperature than HfO_2_. However, both the HfO_2_/Al_2_O_3_ and HfAlO_x_-gated MIS-HEMTs still showed low transconductance, high interface state density and pronounced current collapse. The energy band alignment of MOCVD-HfAlO to GaN was investigated by Liu et al. [175,176], reporting a conduction band offset of 2.2 eV and minimum values of interface trap density in the range of 1–3 × 10^11^ cm^−2^ eV^−1^ at the HfAlO/GaN interface. Hatano et al. [177] demonstrated reduced gate leakage and the improved operation and thermal stability of AlGaN/GaN MIS-HEMTs using a ZrO_2_/Al_2_O_3_ gate stack dielectric.

Other approaches based on the use of Al_2_O_3_-based composite materials have also been reported. Partida-Manzanera et al. [178] investigated the potential of a ternary phase of Ta_2_O_5_ and Al_2_O_3_ as gate dielectrics to achieve higher permittivity than Al_2_O_3_, and hence enhance the transconductance of AlGaN/GaN MIS-HEMTs. Although a higher transconductance and reduced gate leakage current were achieved, the C–V curves did not feature the characteristic step at the forward bias in the spill-over regime, indicating a high density of trap states at the dielectric/AlGaN interface. Kikuta et al. [179] applied Al_2_O_3_/SiO_2_ nanolaminate films deposited by ALD on GaN to obtain a gate dielectric material with a larger conduction band offset to GaN and a higher crystallization temperature than pure Al_2_O_3_ films in order to reduce gate leakage currents. The composition of Al and Si in the oxide and the resulting oxide properties of the permittivity, breakdown field and leakage currents could be controlled and tuned by the numbers of ALD cycles. Compared to pure Al_2_O_3_ films, a higher breakdown field and better reliability were obtained for the SiO_2_ composition, from 0.21 to 0.69. Similarly, Mitrovic et al. [180] suggested that Al_2_O_3_/TiO_2_ nanolaminates can also be favorable as gate dielectrics, and they very recently investigated the band alignment to the GaN and the permittivity of the Al_2_O_3_ layers doped with Ti, corresponding to Ti_x_Al_1−x_O_y_. Although the permittivity of Ti_x_Al_1−x_O_y_ increased significantly with the increasing Ti content, a small conduction band offset for all compositions was obtained. However, Le et al. [181,182] reported excellent characteristics with good insulating properties for MIS-HEMTs using AlTiO deposited by ALD as a gate dielectric.

Current research has also focused on the “doping” by fluorine ions (F^−^) of Al_2_O_3_ gate dielectric films in order to control the threshold voltage of MIS-HEMTs towards normally off operation [183,184]. The latter can be obtained by implanting F^−^ ions into the AlGaN barrier prior to the dielectric ALD. After the ALD-Al_2_O_3_ deposition, the incorporated F^−^ ions can act as a source of negative fixed charges, compensating the intrinsic positive charges in the dielectric and shifting the V_th_ of the device in positive bias direction. It is worth mentioning that a previous physical approach based on the fluorine incorporation via plasma etching under the gate to shift the device threshold voltage was demonstrated by Cai et al. [185]. Using an ALD-Al_2_O_3_ gate dielectric combined with a fluorine-based plasma treatment, Chu et al. [186] demonstrated normally off Al_2_O_3_-gated MIS-HEMTs with a breakdown voltage of 1200 V.

An interesting process was used by Liu et al. [79] and Yang et al. [187], who improved the performance and the V_th_ stability of the Al_2_O_3_-gated hybrid MIS-FETs by inserting a monocrystalline AlN interfacial layer via plasma-enhanced atomic layer deposition (PEALD) at the Al_2_O_3_/GaN interface to block oxygen from the GaN surface and prevent the formation of oxygen-related interface traps. Al_2_O_3_/AlN/GaN structures showed a small frequency dispersion in the C–V curves and a D_it_ in the range of 10^11^–10^12^ cm^−2^ eV^−1^, determined using the conventional conductance method. Similarly, Yang et al. [188] and Chen et al. [189] used an in situ low-damage plasma treatment based on NH_3_ and N_2_ prior to the ALD-Al_2_O_3_ deposition to effectively remove the native oxide while forming an ultrathin monocrystal-like nitridation interlayer (NIL) at the Al_2_O_3_/GaN interface. The N_2_ plasma treatment was also demonstrated to compensate for V_N_-related defects at the surface. After a PDA was carried out at 500 °C in O_2_ ambient, the Al_2_O_3_/NIL-gated MIS structures showed a lower interface trap density in the range of 1–6 × 10^12^ cm^−2^ eV^−1^, resulting in AlGaN/GaN MIS-HEMTs with improved performance [189].

**Figure 7:**
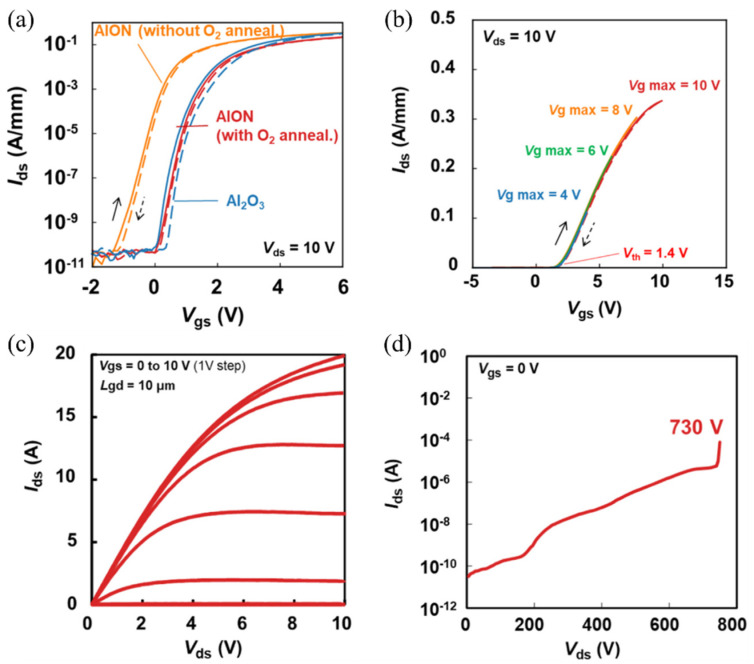
Transfer and output characteristics of recessed-gate AlGaN/GaN MIS-HEMTs using AlON as gate dielectric and subjected to PDA in O_2_ atmosphere, reported by Ueda et al. [192]. The positive shift of V_th_ obtained by O_2_ annealing for the AlON-gated transistor is shown in (**a**), while (**b**) reports the transfer curves without hysteresis obtained after applying a maximum gate voltage up to 10 V. The output characteristics of AlON-gated MIS-HEMTs in the on-state and off-state are shown in (**c**,**d**), respectively.

Finally, a very promising approach proposed by Asahara et al. [190] consists in using a sputtered AlON film as a gate dielectric, obtained by introducing nitrogen into Al_2_O_3_. An atomically abrupt high quality AlON/AlGaN interface with extremely low D_it_ values ranging from 1.2 to 1.4 × 10^11^ cm^−2^ eV^−1^ and improved bulk properties were achieved, resulting in excellent C–V characteristics with negligible frequency dispersions and a markedly suppressed gate leakage current. Similar results were obtained by Wang et al. [191], who deposited AlON films by inserting thin AlN alternating layers into Al_2_O_3_. As shown in Figure 7, Ueda et al. [192] very recently applied AlON films deposited by ALD combined to a PDA in O_2_ for shifting the V_th_ so to realize the normally off operation in the recessed-gate AlGaN/GaN MIS-HEMTs, with a negligible hysteresis in the transfer characteristics, a reduced off-state leakage current, a breakdown voltage of 730 V, an on-state resistance of 270 mΩ for a 10 A drain current rating and impressive switching performance, indicating the great potential of AlON as gate dielectric technology.

## 4. Nitride-Based Dielectrics

Despite the potentiality of Al_2_O_3_ and Al_2_O_3_-based dielectric materials, other insulators have emerged as suitable candidates for insulated-gate GaN-based transistors [1,29]. Among them, nitride-based dielectrics are of particular interest compared to oxide-based insulators because of the suppression of the Ga-O bonds that tend to induce interface traps [187].

SiN_x_ deposited by in situ MOCVD or LPCVD has been widely demonstrated to be very promising both as a gate dielectric and a surface passivation [29,49]. In particular, in situ SiN_x_ enables the dielectric deposition without exposing the (Al)GaN surface to air, which prevents the oxidation of the surface and passivates the surface states, possibly reducing the interface traps. Ogawa et al. [193] demonstrated that the in situ process of SiN_x_ can realize an oxide free SiN_x_/AlGaN interface. Takizawa et al. [194] reported high-resolution TEM analysis revealing abrupt interfaces between SiN_x_ and AlGaN. Jiang et al. [78] systematically investigated MIS structures and MIS-HEMTs using in situ MOCVD-SiN_x_ as a gate dielectric. A D_it_ in the range of 2–3 × 10^12^ cm^−2^ eV^−1^ was obtained, which resulted in a stable V_th_ under gate bias and thermal stress. Derluyn et al. [195] reported that the reduction of surface states with in situ SiN_x_ passivation of HEMT structures led to higher 2DEG density and lower current collapse. Moens et al. [196] even reported on MIS-HEMTs for 650 V applications with excellent interface quality and dielectric reliability using MOCVD-grown in situ SiN_x_, which demonstrated a maximum gate voltage of ~3.1 V at 10 years for a 100 ppm failure rate. LPCVD-SiN has the advantages of a large conduction band offset to GaN (~2.3 eV), a relatively high dielectric constant (~7) and a low defects density enabled by the high deposition temperature. Moreover, compared to plasma-enhanced chemical vapor deposition (PECVD)-SiN_x_, LPCVD-SiN_x_ is free of plasma-induced damage and exhibits low oxygen contamination. In this regard, Hua et al. [197] reported on the superior properties of LPCVD-SiN_x_ in terms of the leakage currents, breakdown field and TDDB lifetime. Similar investigations were performed by Jauss et al. [198], who predicted a 20-year 100 ppm lifetime at 130 °C for a gate voltage of 10.1 V. However, the high deposition temperature of more than 700 °C for LPCVD-SiN_x_ can instead degrade the GaN surface in recessed-gate structures employed for normally off operations [48]. To overcome this issue, Hua et al. [48] successfully employed an interface protection technique consisting of a SiN_x_ interface layer deposited by PECVD prior to the high-temperature deposition process of LPCVD-SiN_x_. With this approach, normally off hybrid MIS-FETs using high-quality LPCVD-SiN_x_ with a gate breakdown voltage of 21 V, a maximum gate bias of 11 V at failure rate of 63.2% for a 10-year lifetime, a stable V_th_ and a small current collapse were demonstrated. A similar approach has been also applied by Jiang et al. [78], who instead used in situ SiN_x_ in conjunction with PECVD SiN_x_ as a passivation scheme to effectively suppress the current collapse in MIS-HEMTs. Finally, it is worth mentioning that for normally off hybrid MIS-FETs, Hue et al. [42] recently developed another promising technique to protect the etched-GaN surface during the LPCVD-SiN_x_ high temperature deposition. This is based on an oxygen-plasma treatment followed by in situ annealing prior to the LPCVD to form a sharp and stable crystalline oxidation interlayer (COIL) protecting the surface. LPCVD-SiN_x_-gated hybrid MIS-FETs with a COIL revealed a stable V_th_ and a highly reliable gate dielectric.

AlN is another promising nitride-based material which is attractive as a gate dielectric for insulated-gate GaN-based transistors due to its large bandgap, resulting in a high breakdown field, high permittivity and small mismatch to GaN, which might reduce the trap states at the AlN/(Al)GaN interface. AlN is mainly grown by MOCVD or PEALD techniques [199]. Hashizume et al. [200] were the first to report the low values of D_it_ in the range of 1 × 10^11^ cm^−2^ eV^−1^ at the MOCVD-AlN/GaN interface. Huang et al. [81] revealed an atomically sharp interface between the PEALD-AlN and AlGaN. They also demonstrated that polarization charges in the monocrystal-like AlN used as a passivation layer can effectively compensate the interface traps at the AlN/(Al)GaN interface, significantly reducing current collapse and the on-resistance degradation in ALD-AlN-passivated AlGaN/GaN HEMTs. Polarization charges in monocrystalline thin AlN layers have also been reported to affect the V_th_ of hybrid MIS-FETs [79]. The high thermal conductivity of AlN has been also shown to be beneficial to suppress the self-heating of AlN-passivated HEMTs, thus improving the device performance [80]. AlN as passivation layer has also been demonstrated to improve the breakdown voltage of AlGaN/GaN HEMTs compared to SiN-passivated devices [201]. Very recently, Hwang et al. [202] reported a sharp interface between the GaN and PEALD-AlN. With the PEALD-AlN used as interfacial layer, they also successfully suppressed the surface oxidation of the GaN, which resulted in the improved C–V characteristics of AlN/GaN structures. AlGaN/GaN MIS-HEMTs and MIS structures using AlN deposited by a novel technique called low-temperature epitaxy (LTE) have been also recently investigated [199,203,204].

## 5. Summary

In this paper, we have summarized the most relevant challenges and recent progress on the development of a gate dielectric technology for insulated-gate GaN-based devices for high-frequency and high-power applications. Specifically, we first pointed out the important physical properties of the insulators which need to be considered for designing a MIS gate structure which delivers improved energy efficiency and reliable device performance. Afterwards, we highlighted that, regardless of the GaN transistor concept and the design, one of the major challenges arising from the insertion of a dielectric on (Al)GaN is represented by the trap states located at the dielectric/(Al)GaN interface or within the bulk dielectric. These trap states strongly affect the performance and the reliability of the device and need to be minimized to ensure high energy efficiency, safe operation and the long-term lifetime of the insulated-gate GaN-based transistors.

Among the various dielectrics, we focused our attention on Al_2_O_3_, which is one of the most promising dielectric materials due to its large bandgap and conduction band offset to (Al)GaN, its relatively high dielectric constant, its high breakdown electric field and its thermal and chemical stability against (Al)GaN. In particular, we pointed out that despite the technological progress in the ALD process, enabling the fabrication of high-quality Al_2_O_3_ films and of Al_2_O_3_-gated devices with improved and reliable performance, a large amount of defects and trap states at the Al_2_O_3_/(Al)GaN interface is still present and still degrades the device performance. In this regard, the main results obtained in the literature of the interface state density distribution at the Al_2_O_3_/(Al)GaN interface are presented and discussed in detail, and the recent progress in the performance of the Al_2_O_3_-gated MIS-HEMTs are reviewed.

Finally, novel Al_2_O_3_-based dielectric or compound materials and interface engineering approaches involving the use of Al_2_O_3_, which have been exploited to improve the quality and electrical performance of Al_2_O_3_-gate MIS structures, have been presented. Among them, AlON, or the use of nitride-based interface control layers have been demonstrated to be the most promising techniques. In addition to that, nitride-based dielectric materials have also been briefly presented as promising candidates, especially driven by their potential to function both as a gate dielectric as well as a passivation layer.

The insights of this paper help to understand the current status and the recent progress of the Al_2_O_3_ gate dielectric technology for insulated-gate GaN-based transistors. It also highlights that the current state of the art has made great advancements, but still requires remarkable progress in terms of gate dielectric, gate stack engineering and interface control technology. Focused efforts are still needed in order to ensure a low interface and bulk trap density, thus enabling a robust reliability under stringent and dynamic electrical stresses. Further advances in the gate dielectric technologies are necessary to overcome these obstacles and to pave the way for the massive advent of insulated-gate GaN-based technologies in the electronic market.

## Data Availability

No new data were created or analyzed in this study.
